# Growth and Development of Children with Microcephaly Associated with Congenital Zika Virus Syndrome in Brazil

**DOI:** 10.3390/ijerph15091990

**Published:** 2018-09-13

**Authors:** Thaís Lorena Barbosa de França, Wilton Rodrigues Medeiros, Nilba Lima de Souza, Egmar Longo, Silvana Alves Pereira, Thamyris Barbosa de Oliveira França, Klayton Galante Sousa

**Affiliations:** 1 Collective Health PostGraduate Program, Faculty of Health Sciences of Trairi, Federal University of Rio Grande do Norte, Santa Cruz 59200-000, Brazil; thais-lorena@hotmail.com (T.L.B.d.F.); wilton.medeiros@ebserh.gov.br (W.R.M.); egmarlongo@yahoo.es (E.L.); apsilvana@gmail.com (S.A.P.); 2 Ana Bezerra University Hospital , Federal University of Rio Grande do Norte, Santa Cruz 59200-000, Brazil; 3 Nursing Graduate Program, Federal University of Rio Grande do Norte, Natal 59072-970, Brazil; nilba.lima@hotmail.com; 4 Rehabilitation Sciences Postgraduate Program, Faculty of Health Sciences of Trairi, Federal University of Rio Grande do Norte, Santa Cruz 59200-000, Brazil; 5 Department of Physical Therapy, Federal University of Rio Grande do Norte, Natal 59072-970, Brazil; 6 City Hall of Touros, Department of Health, Primary Health Care, Touros 59584-000, Brazil; thamyris68@gmail.com

**Keywords:** microcephaly, zika virus, child development, global health, public health, public health surveillance

## Abstract

The outbreak of Zika virus in Latin America in the period 2015–2016 has caused a sudden increase in the number of severe manifestations and reports of congenital changes in newborns in Brazil. This is the first study that evaluated and compared the growth and cognitive and motor development of children with microcephaly due to Congenital Zika Virus Syndrome (CZS) in relation to typical children. It was an observational, analytical, cross-sectional study with 8 children with CZS and 16 typical children, with a mean age of 20.5 months (±2.1), in a region of northeastern Brazil. Considering the mean, children with CZS presented extremely low performance in the motor domain and in the cognitive development domain, whereas typical children presented average performance in the cognitive and motor development domains. Children with CZS presented a mean growth rate (head circumference and weight) lower than typical children. Therefore, children with CZS are at risk for growth retardation and development compared to typical children.

## 1. Introduction

The outbreak of Zika virus (ZIKV) in Brazil caused a sudden increase in the number of severe manifestations and cases of congenital changes in newborns associated with a significant increase in microcephaly reports, especially in the Northeast region [1,2,3,4]. On 1 February 2016, the World Health Organization (WHO) classified this event as a public health emergency of international importance in maintaining itself as such for a period of 9 months [5,6,7,8].

The coefficient of prevalence of microcephaly at birth in Brazil reached 54.6 cases per 100.000 live births (NV) in 2015. The region with the highest coefficient was the Northeast (139 cases per 100 thousand NV), which corresponds to 28 times the annual average coefficients for that region in the period 2000–2014 (5.0 cases per 100.000 NV) [9].

Several studies presented evidence that convinced the Brazilian Ministry of Health to acknowledge the relationship between the presence of ZIKV and the occurrence of microcephaly. Among the evidence reported, there is evidence that the virus crosses the placental barrier, presents tropism by the nervous system [10,11,12,13,14], and has a greater circulation in the period equivalent to the first trimester of gestation of women with children with microcephaly [5]. Currently, it is universally understood that microcephaly is not the only finding but a consequence of several brain lesions characterizing this condition as Congenital Zika Virus Syndrome (CZS) [12].

The outbreak of ZIKV in Brazil has receded. However, the social and economic impacts are of a late-arriving and lasting nature [15]. Deficiency and poverty are considered elements that interact with one another in a cycle in which one element reinforces the other. In developing countries, marked by social and economic inequalities, this relationship is perceptible, so, in addition to the vector control carried out by Brazilian public agencies, massive investments in public infrastructure services are needed [16].

It is therefore understood that a continuous, intensified, and interdisciplinary international response is needed to improve the ability to anticipate, control, and mitigate the risk of ZIKV, and other re-emergent arboviruses and emerging public health threats [15] anchored in health promotion and protection practices, which allow for the identification of children who are more vulnerable and at high risk of disease, morbidity, and mortality [17].

In this context, situations of vulnerability of children with CZS are created, with limited health conditions and the low socioeconomic status associated with increasing needs for health services, as a result of their physical and intellectual disabilities [18]. Thus, through the high risk of alterations, it is necessary to permanently monitor the growth and development of these children.

The biological process of child growth and development is considered complex and progressive and is linked to genetic factors and environmental stimuli. In this sense, they are considered to be health indicators for a child and show the life and health conditions of this population. In the complexity of this distinct situation, evaluating and monitoring child growth and development is fundamental for the systematization of behaviors, interventions, and follow-up in public health networks in Brazil [17].

In this regard, WHO recommends the development of disability research in line with the United Nations’ sustainable development agenda and the United Nations Convention on the Rights of Persons with Disabilities [19].

The present investigation is relevant because it is a pioneer in the surveillance approach to child growth and development related to the outbreak of CZS in Brazil; therefore, it also has the characteristic of novelty. It aimed to evaluate the current state of growth and development of children with CZS compared to typical children in a territory of the Brazilian Northeast.

## 2. Materials and Methods

### 2.1. Design

This is an observational, analytical, cross-sectional study with a quantitative approach.

### 2.2. Context

This study was carried out during a master degree in Collective Health at the Federal University of Rio Grande do Norte, Faculty of Health Sciences of Trairi, in a city in the interior of the State of Rio Grande do Norte (RN), Brazil, from August 2016 to April 2018. The subjects of the research were recruited from June 2017 to January 2018 and the data was collected in the period of July 2017 to January 2018.

### 2.3. Participants

All children affected by CZS in this study showed changes in brain morphology detected through imaging tests, such as transfontanel ultrasonography, cranial computed tomography, and/or cranioencephalic magnetic resonance imaging. These exams revealed a reduction in brain volume, alterations in the cerebral ventricles, and cerebral calcifications. Some children in the sample had ophthalmological alterations, especially macula and optic nerve alterations; however, they did not present congenital foot or arthrogryposis.

The children were in a rehabilitation follow-up with attendances in the areas of physical therapy, speech therapy, pediatrics, and ophthalmology in specialized services or by early stimulation of Brazilian public health organs.

In view of the scarcity of serology kits for ZIKV, especially at the beginning of the outbreak, there was no laboratory confirmation of this infection in all of the mothers of the children in this study. However, all the children in the sample who were born with congenital abnormalities were considered to be confirmed cases of microcephaly due to ZIKV infection based on the criteria of the Brazilian public health confirmatory organs and according to the protocol for the investigation of cases of microcephaly [20].

The protocol chose as confirmatory criteria the following: Ultrasonographic finding of fetus with alteration in the central nervous system (CNS) suggestive of congenital infection, reporting exanthema in the mother during pregnancy, excluding other possible infectious and noninfectious causes or conclusive laboratory diagnosis for Zika virus; Ultrasonographic finding of fetus with measured cranial circumference less than two standard deviations (<2 dp) below the mean for gestational age with or without other CNS changes, excluding other possible infectious and noninfectious causes or with conclusive laboratory diagnosis for Zika virus; Ultrasonographic finding of fetus with CNS alteration suggestive of congenital infection, reporting exanthema in the mother during pregnancy, excluding other possible infectious and noninfectious causes or with conclusive laboratory diagnosis for Zika virus [20].

**Figure 1:**
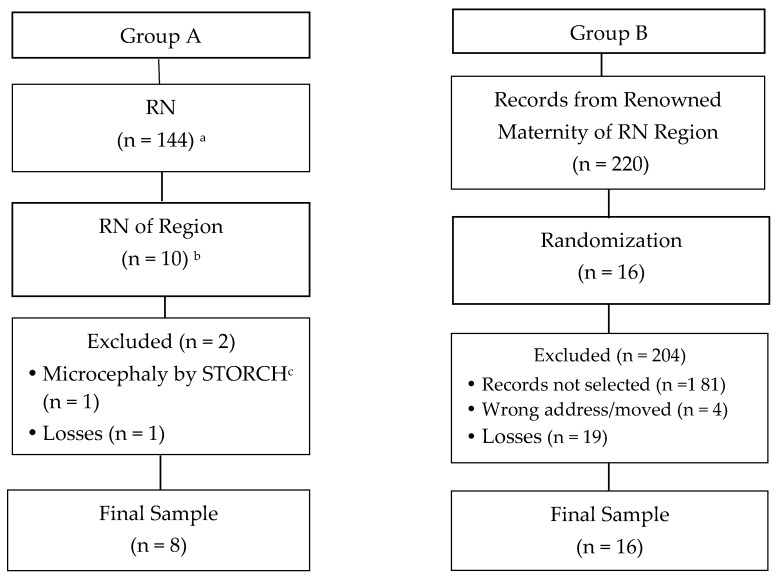
Flow diagram of subject recruitment for the research study. ^a^ Confirmed cases of microcephaly and/or other malformations related to congenital infections during April 2017 in State of Rio Grande do Norte of Brazil (RN). ^b^ Cases of microcephaly related to Zika virus (ZIKV). ^c^ STORCH: Syphilis, Toxoplasmosis, Rubella, Cytomegalovirus, Herpes.

The study included children with a mean age of 20.5 months (±2.13) living in the territory of a Health Unit of the State of Rio Grande do Norte and divided into two groups according to Figure 1. The inclusion criteria for group A were children presenting CZS microcephaly and residing in the selected territory. In group B, the inclusion criteria were children who did not present CZS and microcephaly of any origin nor neurological pathologies, who resided in the municipality of Santa Cruz, state of Rio Grande do Norte, and whose respective mothers did not present during pregnancy any STORCH (Syphilis, Toxoplasmosis, Rubella, Cytomegalovirus, Herpes) diseases. Exclusion criteria were physical and/or mental illness of the guardian or other condition that would impair evaluation and care of the child during the study.

In group A, the possibility of infection with other flaviviruses was excluded due to the investigation carried out in Brazilian centers specialized in laboratory diagnoses. In group B, this exclusion occurred due to the absence of arbovirus infections during the gestational period of these mothers verified through hospital records and the prenatal notes in the mother’s health records notebook of the Brazilian Ministry of Health associated with the absence of clinical findings during the development of children in this group.

Participants in group A were recruited through a personal address contact provided by the public health organs of the region upon prior consent. All children in the CZS region were recruited (n = 10). During the study, two (n = 2) children were excluded due to a change of diagnosis or change of address and/or telephone contact, making it impossible to contact them; the final total was eight children (n = 8). The sample of group B was of the probabilistic type, simple random, and composed of 16 children matched by gender and age range with the children of group A in the ratio of 1:2.

The formation of group B was performed in two stages. In the first stage, eight lists of 10 children were constructed. Information on the lists (mother’s name, address and telephone contact) were provided by records from the maternity hospital renowned in the region after consent from the institution was obtained. Then, a simple draw of two children was carried out over each of the lists until the formation of group B was finished (n = 16).

Further draws were made when contact with the person responsible for the child could not be obtained through the residential address and/or telephone contact available in the hospital records, as well as when the responsible person was not authorized to participate in the study or in cases of withdrawals.

### 2.4. Equipments and Test Instruments

The growth variable was measured by weight, body length, and cephalic perimeter using as reference standard the Z score. This score is used to represent the variability of a given parameter among individuals and represents the distance, in standard deviation, that the values of that parameter can assume in the population in relation to the average value [21].

Weight was evaluated on a precision, calibrated, and platform-type digital scale. Children who did not remain in the orthostatic position without assistance were assessed by the difference in weight of the set (adult weight plus child weight minus adult weight). The length of the child was measured through a horizontal stadiometer and the cephalic perimeter through an inelastic tape measure at the height of the supraorbital arches anteriorly and in the greater prominence of the occipital bone posteriorly. A questionnaire containing questions about the social and demographic conditions of the family nucleus of the research subjects was also applied.

A child’s cognitive and motor development was measured through the Scales of Infant and Toddler Development Third Edition (Bayley-III) with all their standardized and original material and following the guidelines of their manuals. The scale was applied in the Portuguese language through a free translation by a team trained by the research organizer.

Bayley-III is a tool of North American origin used to evaluate the development of children aged 15 days to 42 months. Its third edition was published in 2006 and is a revision of the second edition released in 1993. It is composed of cognitive sub-receptive language, expressive language, fine motor skills, gross motor skills, and social-emotional and adaptive behavior. In the third edition, there was an update of the normative data and advances that ensured better psychometric quality, greater facilitation of clinical use, and an improvement in normative characteristics [22,23].

It is a widely used evaluation instrument and considered as the gold standard, or reference standard, in children’s health. It is a diagnostic tool that can identify possible delays in child development due to its solid theoretical foundation and robust psychometric properties [22,24,25,26].

This scale also helps to plan interventions in important clinical settings and helps to understand the strengths and weaknesses of the child in all five domains of development. However, it is known that results from its application should not be interpreted in isolation [22,25]. We highlight a study carried out in nurseries in a city of Greater São Paulo that culturally adapted and validated the Brazilian version of Bayley-III. This version had high convergent validity and good internal consistency and homogeneity for children aged 12 to 42 months [24].

According to Bayley-III, development is classified by scores, using standard scores for a composite score, ranging from 40 to 160 points across all of its subscales. The deviation of an individual’s score from the normative mean (100 ± 15) is used to classify developmental delay [23,26].

In this sense, considering the possible variations in the scale above or below the reference average, the scale recommends that performance be indicated as very superior (score 130 or above), superior (score between 120 and 129), high average (score between 90 and 119), average (score between 90 and 109), low average (score between 80 and 89 points), borderline (score between 70 and 89), and extremely low (score 69 or below). We emphasize that overall motor performance is the result of fine motor performance added to gross motor performance [27,28].

### 2.5. Data Collection Procedures

This study complied with Resolution No. 466/12 of the National Health Council (Brazil) and was approved by the Research Ethics Committee of Faculty of Health Sciences of Trairi (FACISA) with the number of opinion 1.839.750 and CAAE 58715316.3.0000.5568. All procedures were carried out after consent was obtained from the family member responsible for the child through the signing of the Informed Consent Form and the Authorization Term for the use of Images and Videos.

After the agreement of the parents or guardians of the child and through their availability, a single evaluation was scheduled. Initially, data were collected on the birth of the children from the records at the Maternity Hospital in the territory studied and, in the absence of this document, information in the child’s booklet was recorded. This booklet belongs to the family of the child and contains all the child’s medical information and their vaccination status after birth.

The data collection of children of Group A happened at the Physical Therapy Clinic of FACISA where the children are assessed periodically and are accustomed to the environment. For convenience, children in Group B were evaluated at their home. The collection was canceled if the child was ill or at the request of the parents. Assessment sessions were recorded using a digital video camera and scores were recorded on Bayley-III’s own form after reviewing the footage.

### 2.6. Statistical Analysis

The descriptive variables related to the general characteristics of the children were ethnicity, gestational age, type of delivery, and apgar, and those related to mothers were maternal age, place of residence, marital status, family income, education, and profession. These variables were presented in the form of absolute numbers: percentage, median or mean and standard deviation.

The independent variables of this study were measures of weight, body length, and cephalic perimeter that are markers of child growth besides the composite performance of cognitive and motor development. The statistical analysis of these variables was based on the comparison of independent groups paired by gender and age range by applying the non-parametric Mann–Whitney statistical test in the SPSS version 20.0 software for Windows 8 (IBM, Armonk, NY, USA) with the significance level of 5% and the test power of 95%.

## 3. Results

The descriptive analysis of the sample of this study was performed by calculating the absolute frequency and percentage. In the present study, the majority of children in group A were white, born through vaginal delivery, full-term, with satisfactory values of apgar index in the first and fifth minutes of life, and with a mean age of 21.14 months. In group B, the majority of the children were white, born of cesarean birth at term, with satisfactory values of apgar index in the first and fifth minutes of life, and with an average age of 20.48 months. Regarding the maternal characteristics, we found that in group A, the majority of mothers had a mean age of 25 years, lived in an urban area, lived together in a stable union, had incomplete basic education, were women farmers, and had low family income. In group B, the majority of the mothers had a mean age of 28 years, lived in an urban area, were married, had a high average educational level, and were housewives with low family income (Table 1).

**Figure 2:**
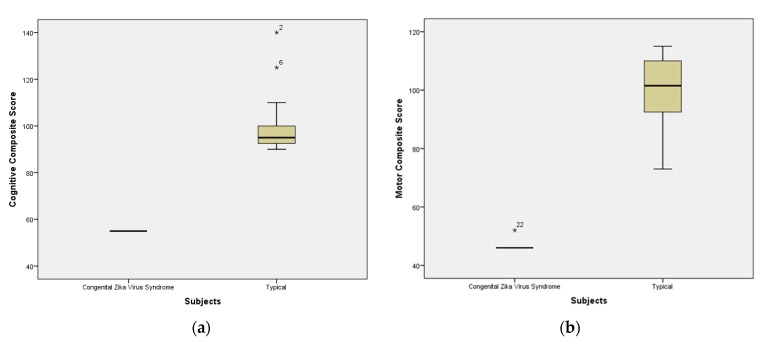
(**a**) Cognitive Composite Score divided by groups; (**b**) Motor composite score divided by groups (n = 8 group A; n = 16 group B); * Represent the outliers of sample.

The distribution of children, according to cognitive and motor composite performance, is described in Figure 2. Considering the classification recommended by Bayley-III, group A participants presented a mean of motor and cognitive performance below the minimum limit of the average (90) and the reference range (100), representing developmental delay of the extremely low type in the cognitive domain (mean 55 ± 0) as well as in the motor domain (47 ± 2).

Children in Group B presented normal development in the cognitive domain (100 ± 14) as well as in the motor domain (100 ± 12). The performance comparison between the cognitive and motor domains of the two groups (Mann–Whitney U test) showed a significant difference (*p* = 0.000). The cognitive domain had U = 0.000 and z = −4.038 and the global motor domain had U = 0.000 and z = −3.974.

Regarding infant growth (Table 2) and taking into account the z score of the anthropometric measurements of birth records in both groups, it was noted that the children of Group A were born with adequate measures, except for the cephalic perimeter (mean of the score z = −2.3), but in the evaluation performed in this study, with an average age of 20.5 months, Group A children did not present adequate measures according to their peers. Thus, we found a significant difference (*p* < 0.05) in weight measurements (U = 15.500, z = −2.971) and cephalic perimeter (U = 0.000, z = -3.929) among the groups of children evaluated.

## 4. Discussion

This study evaluated children growth through anthropometric measures and development through the cognitive and motor domains of children with CZS microcephaly compared to typical children at the mean age of 20.5 months. In this study, children with CZS presented lower performance in the domains evaluated than the typical children, and this difference had statistical significance.

Regarding the anthropometric measurements of weight, length, and cephalic perimeter, there was a statistically significant difference between the groups regarding head circumference and body weight in the evaluation. This finding validates the understanding that children with CZS remained with microcephaly when compared to their peers (typical children) and associated with this, it was observed that there was no adequate weight gain for the age group, which was ratified by the statistically significant difference in body weight.

A statistically significant difference was elicited in the birth measurements taken from the birth registry of the children and from the cephalic perimeter; however, the same did not happen with the weight and length measurements. This reinforces the fact that the children were born with the weight and length compatible with and adapted to their gestational ages.

The results described agree with the findings of the Bayley-III normative sample [22,23], applied to 73 children aged 5 to 42 months with cerebral palsy (CP), since motor skills scores were significantly lower in children with CP than in the combined control group and there was a low mean in the cognitive score in the group of children with CP.

In Brazil, a national profile was published in 2017 of mothers of children born with CZS, which indicated that 71% of mothers lived in the Northeast, 51% were up to 24 years of age, 77% were of mixed race or black, and 60% had eight or more years of formal education. This author also affirms that the profile presented shows the socioeconomic and geographical inequalities of the families of the children who present with the complaint [9]. There was similarity in the predominance of the younger maternal age group and in schooling.

There was agreement with a Dutch study in which 77 children with intellectual disabilities and typical children aged 1 to 9.10 years had a cognitive domain with a mean of 39.8 (±18) and 103 (±15) for children with and without disabilities, respectively [28]. This information reinforces the understanding that neurological changes, such as those resulting from cerebral palsy and other abnormalities, compromise cognition even though the more marked impairment of motor development is more common.

Contrary to what was presented in this study, Amir et al. (2016) found that in only 6% of children with cytomegalovirus the finding of microcephaly was present and those children presented cognitive performance within normality [29]. This study included 27 children aged 1 to 3 years. With this, we can infer that CZS probably has a marked severity compared to cytomegalovirus congenital syndrome.

Araujo et al. (2016) reported a case-control study in the State of Pernambuco, Brazil with 32 newborn cases and 62 controls. The newborns in the case group had microcephaly. These authors reported that 94% of the control group had adequate weight, whereas only 16% of the children with CZS presented this finding. We also identified abnormal brain imaging findings in 41% of newborns [13]. It is important to point out that, unlike our study, it presented a large proportion of small infants for gestational age.

Differently from these last-mentioned studies, the study Zonta et al. [30] with children with hemiplegic paralysis aged 3 to 5 years indicated averages of weight, height, and cephalic perimeter that were adequate for age with 21% of the finding of microcephaly.

A Brazilian study revealed the functional profile, based on the International Classification of Functioning, Disability, and Health (ICF), of children with microcephaly by ZIKV in rehabilitation centers of the Brazilian states. This study found complete disability in most categories of body functions with an important impact in the areas of activity and participation and in the categories related to mobility. With regard to environmental factors, the majority of the sample reported immediate family, friends, and health services, systems, and policies as facilitators and pointed to social attitudes as a barrier [31].

The sample size is a limitation of our study and therefore we emphasize that our results are not generalizable, but they portray real situations of retardation in the growth and development of a group of children with microcephaly resulting from ZIKV, which can be found in other groups with similar characteristics.

We have shown the need for a specialized care network for these children, especially with early stimulation. We also point out that new research, especially longitudinal research, is needed on child growth and development aimed at monitoring the health of this vulnerable population.

## 5. Conclusions

The family context in which children with CZS are inserted alerts us to a situation of individual and social vulnerability in which children are exposed to serious health problems and family economic fragility. In this study, these children were found to be at risk for motor and cognitive developmental delay and growth decline compared to typical children of the same age. The challenge is for public policies to ensure that children with disabilities can participate in daily and leisure activities under the same conditions as their non-disabled peers, which will contribute to increasing their motor and psychosocial skills. Investing in interventions focused on minimizing barriers to the physical, social, and attitudinal environment can be an effective strategy, especially in low- and middle-income settings.

## Figures and Tables

**Table 1:** Average, Standard Deviation, and percentage distribution by categories in relation to sociodemographic/clinical variables of the children and sociodemographics of the mothers studied (n = 8 group A; n = 16 group B).

Characteristics of Children		
	Group A * (n = 8)	Group B ** (n = 16)
Age (months)		
Average	21.14 ± 1.95	20.48 ± 2.16
Ethnicity		
White	62.50%	62.50%
Mixed	37.50%	37.50%
Gestational age at birth		
Full-term	85.70%	100%
Post-term	14.30%	0%
Type of Delivery		
Normal	75%	31.20%
Cesarian Section	25%	68.80%
Apgar 1’		
Median	9	9
Apgar 5’		
Median	9	9
**Characteristics of Mothers**		
Age (Year)		
Average	25 ± 6.45	28 ± 7.52
Residence		
Urban Area	75%	100%
Countryside	25%	0%
Marital Status		
Single	25%	6.20%
Married	25%	56.20%
Stable Union	50%	37.50%
Education		
Incomplete Elementary Education	50%	12.50%
Complete Elementary Education	0%	18.80%
Incomplete Secondary Education	0%	18.80%
Complete Secondary Education	37.50%	37.50%
Complete University Education	12.50%	12.50%
Profession		
Housewife	37.50%	56.20%
Farmer	50%	0%
Others	12.50%	43.80%
Gross Annual Income		
<US$10,000	87.50%	81.30%
US$10,000–15,000	12.50%	18.70%

* Group A: Children with congenital ZIKV syndrome. ** Group B: children with normal development.

**Table 2:** Average, standard deviation, and significant differences between groups in relation to infant growth (n = 24).

Variable (Average)	Group A (Children with CZS *)	Group B (Typical Children)	*p* Value **
**At Birth**			
Weight	2.8 ± 0.4	3.2 ± 0.6	*p* = 0.153
Length	45.9 ± 3.6	48.6 ± 2.1	*p* = 0.065
Cephalic Perimeter	31.0 ± 1.4	34.6 ± 1.1	*p* = 0.000
**At Assessment**			
Weight	9.1 ± 1.0	11.8 ± 1.9	*p* = 0.002
Length	77.9 ± 3.4	85.2 ±5.6	*p* = 0.038
Cephalic Perimeter	40.2 ± 2.2	47.3 ± 1.5	*p* = 0.000

* Congenital Zika Virus Syndrome (CZS)/**Test Mann–Whitney, *p* < 0.05.
